# Shear Performance at Room and High Temperatures of Glass–Ceramic Sealants for Solid Oxide Electrolysis Cell Technology

**DOI:** 10.3390/ma12020298

**Published:** 2019-01-18

**Authors:** Hassan Javed, Antonio Gianfranco Sabato, Ivo Dlouhy, Martina Halasova, Enrico Bernardo, Milena Salvo, Kai Herbrig, Christian Walter, Federico Smeacetto

**Affiliations:** 1 Department of Applied Science and Technology, Politecnico di Torino, Corso Duca Degli Abruzzi 24, 10129 Turin, Italy; antonio.sabato@polito.it (A.G.S.); milena.salvo@polito.it (M.S.); 2 Institute of Physics of Materials, Zizkova 22, 61662 Brno, Czech Republic; idlouhy@ipm.cz (I.D.); halasova@ipm.cz (M.H.); 3 Department of Industrial Engineering, Università degli Studi di Padova, via F. Marzolo 9, 35131 Padova, Italy; enrico.bernardo@unipd.it; 4 Sunfire GmbH, Gasanstaltstraße 2, 01237 Dresden, Germany; Kai.Herbrig@sunfire.de (K.H.); Christian.Walter@sunfire.de (C.W.); 5 Department of Energy, Politecnico di Torino, Corso Duca Degli Abruzzi 24, 10129 Turin, Italy; federico.smeacetto@polito.it

**Keywords:** glass–ceramic, shear strength, elastic modulus, SOFC, SOEC

## Abstract

To provide a reliable integration of components within a solid oxide electrolysis cell stack, it is fundamental to evaluate the mechanical properties of the glass–ceramic sealing materials, as well as the stability of the metal–glass–ceramic interface. In this work, the mechanical behavior of two previously developed glass–ceramic sealants joined to Crofer22APU steel is investigated at room temperature, 650 °C, and 850 °C under shear load. The mechanical properties of both the glass–ceramics showed temperature dependence. The shear strength of Crofer22APU/glass–ceramic/Crofer22APU joints ranged from 14.1 MPa (20 °C) to 1.8 MPa (850 °C). The elastic modulus of both glass–ceramics also reduced with temperature. The volume fraction of the crystalline phases in the glass–ceramics was the key factor for controlling the mechanical properties and fracture, especially above the glass-transition temperature.

## 1. Introduction

Solid oxide electrolysis cells (SOECs) are a promising technology to produce hydrogen through the electrolysis of water. Among the various components of an SOEC stack, the stability and performance of the glass–ceramic sealants are key factors to determine and control the overall efficiency of the system [1,2]. Because of the operational thermal regime of the whole stack, the joined components are subjected to the change of the acting stress, from almost compressive and/or tensile to almost shear. In laminate structures, this means that not only the SOEC materials, but also their interfaces often play a crucial role [3,4].

Due to cyclic temperature working conditions, there is a possibility of stress generation at the Crofer22APU/glass–ceramic interface and/or within the glass–ceramic joint. If the stresses increase to a critical level, either debonding at the Crofer22APU/glass–ceramic sealant interface or within the glass–ceramic can occur, thus causing gas leakage. Therefore, besides the thermal, chemical, thermomechanical and electrical stability of glass sealants, it is also important to analyze their response to mechanical loading under conditions corresponding to operational ones [5,6,7,8,9,10]. The high-temperature mechanical behavior of glass–ceramic sealants is crucial due to possible deterioration of these properties, especially if the working temperature is higher than the glass-transition temperature (*T_g_*) [11,12,13]. Recently, a few researchers have studied the mechanical properties of glass–ceramic sealants at room and high temperatures [14,15,16,17,18,19,20,21]. Selçuk et al. [18] employed three different testing methods to investigate the shear strength of glass–ceramic sealants, without specifying the glass-based composition: single lap offset (SLO) under compression, single lap (SL) under compression and asymmetrical 4-point bending test (A4PB). They found a significant variation in the apparent shear strength obtained by the different methods; specifically, the shear strength measured in the SLO configuration was relatively low (around 7 MPa), likely due to significant normal tensile stresses perpendicular to the joint. Stephens et al. [22] and Lin et al. [13] investigated the tensile and shear properties between the Crofer22APU interconnect and glass–ceramic sealants from room temperature to 800 °C. Stephens et al. tested a barium–calcium–aluminosilicate-based glass-sealing material (G18); tensile and torsion tests were performed to characterize the interfacial shear strength between the G18 glass and the Crofer22APU. The mechanical strength of the joint decreased by almost 50% with an increase in temperature from 25 °C to 800 °C [22]. Lin et al. evaluated the joint strength of a BaO–B_2_O_3_–Al_2_O_3_–SiO_2_ glass–ceramic joined to the Crofer22H specimen, at room temperature and at 800 °C under shear and tensile loading. They evaluated the effect of ageing and pre-oxidation of the Crofer22H and found that the tensile joint strength is lower if the fracture involves delamination at the interface between the steel substrate and BaCrO_4_ layer, formed by the reaction between BaO from the glass and Cr from the steel. Anyhow, by increasing the testing temperature, the shear strength reduced from 7 MPa (25 °C) to 4 MPa (800 °C) [13]. 

In a paper by López et al. [23], the flexural strength of two glass–ceramic (one containing Ba and one containing Sr) bars using a three-point bending setup after different ageing times was measured. Although the glass–ceramics were not interfaced with a metallic interconnect, interesting results were obtained in terms of the comparison between the mechanical properties of the two different glass–ceramic compositions. The authors discussed the mechanical behavior regarding different thermal ageing times of the glass–ceramics and their microstructural evolution. The glass–ceramics containing SrO exhibited higher flexural strength than the glass–ceramics with BaO [23]. 

In our work, the shear strength of the Crofer22APU/glass–ceramic/Crofer22APU joined samples was studied by using two SrO-containing glass-based systems, designed for a working temperature of 850 °C. The Crofer22APU/glass–ceramic/Crofer22APU samples were investigated under shear load at room temperature, 650 °C and 850 °C. The elastic modulus of the glass–ceramics was also measured from room temperature to 650 °C by vibration method. 

## 2. Materials and Methods 

Two previously developed glass–ceramic systems, further labeled as HJ3 and HJ4, are employed to investigate their mechanical behavior in contact with the Crofer22APU interconnect. The *T_g_* of the HJ3 and HJ4 glasses was 722 °C and 736 °C, respectively, as measured by differential thermal analysis (DTA). Previously performed XRD analysis showed the presence of Sr_2_Al_2_SiO_7_, Ca_0.75_Sr_0.2_Mg_1.05_(Si_2_O_6_) and Ca_2_Mg(Si_2_O_7_) phases in the HJ3 glass–ceramic, while the HJ4 glass–ceramic had SrSiO_3_ and SiO_2_ phases after joining [24]. 

The coefficients of thermal expansion (CTE) and the softening behavior of both the glass–ceramics were measured by dilatometer (Netzsch, DIL 402 PC/4, Selb, Germany), at a heating rate of 5 °C/min. The dilatometer analyses were performed on the glass–ceramic pellets (diameter 1 cm), prepared by pressing the glass powder in a steel mold, followed by a heat treatment in static air. Quantitative XRD analyses based on the Rietveld method were not feasible for the as-joined HJ3 and HJ4 glass–ceramics due to the complex crystalline phases and the corresponding XRD patterns. Consequently, in order to determine the relative quantities of the crystalline phases in the HJ3 and HJ4 as-joined glass–ceramics, an estimation could be made on the relative weight balance between the crystals in the glass–ceramics and the internal standard (ZnO), introduced in a defined quantity (20 wt.%). Therefore, the semi-quantitative analysis was performed by means of a Match software package (version 1.10, Crystal impact, Bonn, Germany), operating based on the reference intensity ratio method (RIR method) [25]. 

**Figure 1:**
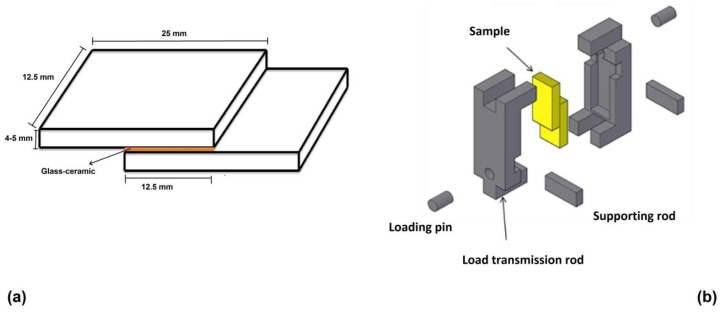
Illustration of Crofer22APU/glass–ceramic/Crofer22APU samples with glass–ceramic joint for shear testing (**a**) and setup for testing the sample under shear load (**b**).

For mechanical characterization under quasistatic shear loading conditions, the Crofer22APU/glass–ceramic/Crofer22APU joined samples were prepared. Figure 1 illustrates the sample configuration (including dimensions) subsequently tested under shear load. Before joining, both plates of Crofer22APU were made plane parallel and polished to obtain the desired dimensions with a tolerance of ± 0.1 mm. Each plate was cleaned by using acetone and subsequently the glass was deposited in the form of a slurry containing glass particles in ethanol (70:30 wt.%). The joining of the HJ3 glass–ceramic with the Crofer22APU was carried out at 950 °C for 1 h at a heating rate of 5 °C/min, while for the HJ4 system the joining was processed at 950 °C for 5 h at a heating rate of 2 °C/min. The glass–ceramics in the joining region had a thickness of 600 µm ± 50. After joining, the samples were again gently polished for a few minutes to make sure that both steel plates were perfectly parallel to each other. 

Quasistatic shear testing was carried out at a constant machine cross-head rate of 50 µm/min. The loading fixture developed for the experiments is also shown in Figure 1. The red arrows in Figure 1 indicate the direction of the applied load. Tests were conducted at three different testing temperatures, namely room temperature, 650 °C and 850 °C. The displacement of the joined plates was quantified by using a high-temperature extensometer located outside the furnace. The Zwick/Roell–Messphysik Kappa 50kN test system with a Maytec inert gas high-temperature chamber was used for the experiments. The joint area of each sample was measured after the shear test by using a light microscope with CCD camera and image analysis. The shear stress was then calculated by dividing the applied load by the real joint area. All tests were conducted in an argon atmosphere. Before each test, the sample was heated to the desired temperature and kept at that temperature for 3 h, to make the temperature homogenous throughout the heating zone of the chamber. The temperature was measured by a thermocouple attached directly to the sample. To obtain statistically representative data, at least three samples of both compositions were tested at each temperature. The post mortem analysis of broken samples was carried out by scanning electron microscope (SEM, Merlin ZEISS, Munich, Germany). For this purpose, cross sections of the Crofer22APU/glass–ceramic interfaces were metallographically polished up to 1 µm by diamond paste and investigated by SEM after being coated with gold. 

The elastic modulus of the pure glass–ceramics was measured by vibration method, at temperatures ranging from room temperature to 650 °C, during the heating and cooling cycles. For this purpose, thin rectangular samples of glass–ceramics with dimensions of 20 mm × 2 mm × 2 mm were prepared. The high-temperature impulse excitation technique, HT1600 system (IMCE, Belgium) was applied for these analyses. The elastic modulus was determined by measuring the resonant frequency of the sample at the given temperature and then calculated from the specimen dimensions and density. 

## 3. Results and Discussion

The previously performed DTA on these glass systems showed a significant difference in crystallization behaviors. No crystallization peak was detected during the DTA analysis of the HJ4 glass, thus indicating that the crystallization was probably not sufficient to be detected. However, the DTA analysis of the HJ3 glass showed a crystallization peak due to the sufficient crystallization in that system [25]. 

**Figure 2:**
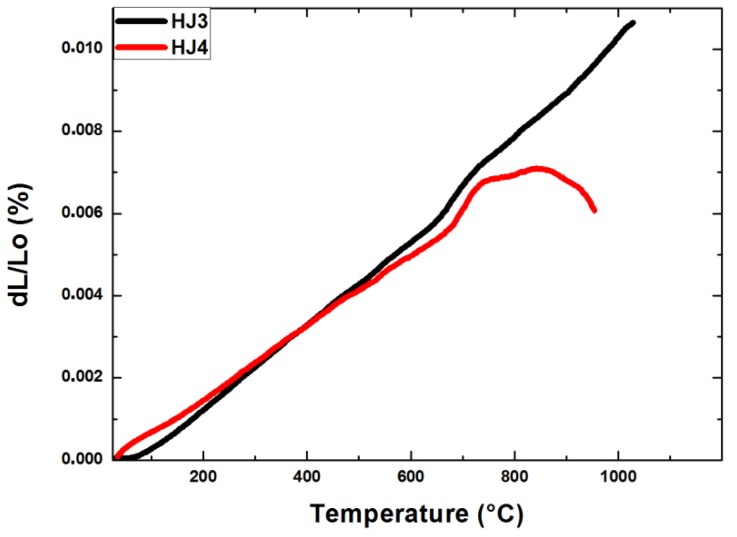
Dilatometer curves of as-joined HJ3 and HJ4 glass–ceramics. Measurements were carried out at a heating rate of 5 °C/min.

The dilatometer curves of the HJ3 and HJ4 glass–ceramics are shown in Figure 2. The CTE of the as-joined HJ3 and HJ4 glass–ceramics were 10.2 × 10^−6^ K^−1^ and 9.3 × 10^−6^ K^−1^ respectively, in the temperature range of 200 °C–500 °C. The dilatometer curve of the HJ3 as-joined glass–ceramic is quite linear, thus indicating that the HJ3 glass–ceramic did not become soft up to 1000 °C. Nevertheless, the dilatometer curve of the HJ3 glass–ceramic shows a slight change in the slope around 680 °C, which is most likely due to the *T_g_* of the residual glassy phase. On the other hand, the dilatometer analysis of the HJ4 glass–ceramic shows that the HJ4 glass–ceramic became soft at around 800 °C with a *T_g_* around 700 °C. These results further support the hypothesis that the as-joined HJ4 glass–ceramic has more residual glass than the as-joined HJ3 glass–ceramic. 

**Figure 3:**
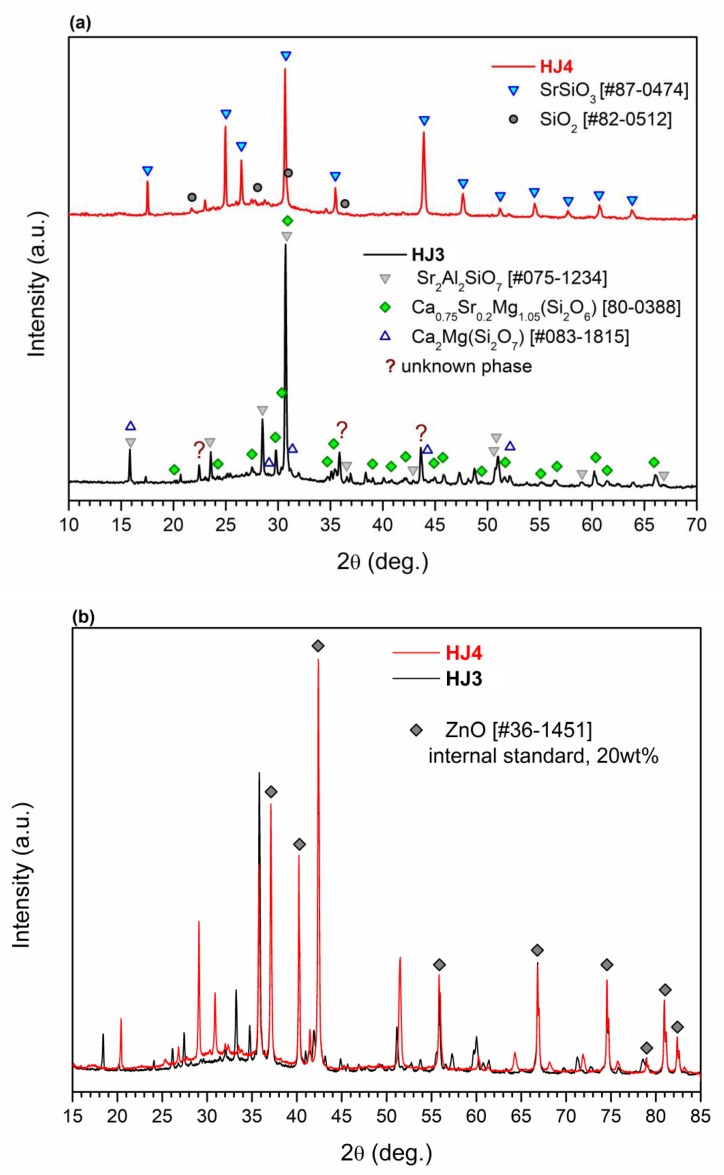
(**a**) Indexed XRD patterns of the HJ3 and HJ4 as-joined glass–ceramics (**b**) XRD pattern of as-joined glass–ceramics and ZnO standard.

Figure 3a shows the XRD analyses performed on the as-joined HJ3 and HJ4 glass–ceramics. Due to the complex crystalline phases (especially in HJ3), some peaks are unidentified, as shown in Figure 3a,b shows the XRD patterns of both glass–ceramics after the inclusion of the standard (ZnO). Closer inspection of the XRD reported in Figure 3b shows the presence of a slightly more pronounced “amorphous halo” in the XRD pattern of the HJ4 glass–ceramic with respect to HJ3 system, thus undoubtedly indicating a relatively lower degree of crystallization in the HJ4 system. Even if the HJ4 system was processed for longer time in comparison with HJ3 (5 h vs. 1 h), it maintained a significant amount of amorphous phase, as further supported by the dilatometer curves of the as-joined HJ3 and HJ4 glass–ceramics shown in Figure 2, thus highlighting a significant difference between the two compositions. These results agree with the results obtained from the DTA, HSM and dilatometer analyses of these glass–ceramics i.e., the HJ4 glass–ceramic has a relatively higher quantity of residual glass than the HJ3 as-joined glass–ceramic.

**Figure 4:**
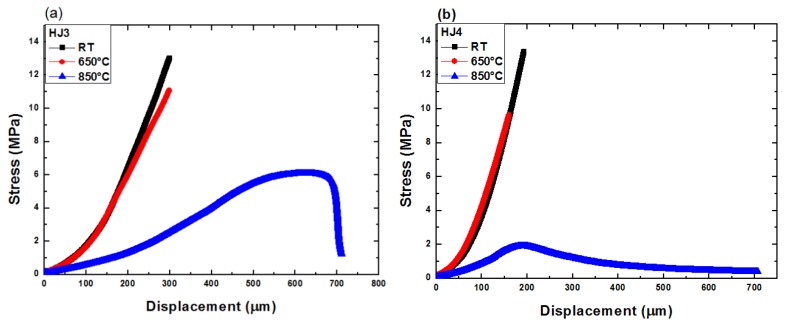
Shear stress vs. displacement curves for (**a**) HJ3 and (**b**) HJ4 joints, tested under shear loads at three different temperatures.

Figure 4 shows the shear stress vs. load displacement curves for the HJ3 and HJ4 joint samples, tested at three different temperatures. For the sake of simplicity, only one curve has been shown for each temperature measurement. The traces reflect how the high temperatures significantly affect the deformation and fracture behavior of the joints made by the two different glass–ceramics. Please note that the slope of the linear part of the curves may be affected by an inexact determination of the joint area, as carried out by post mortem analysis of the fracture surfaces. When testing the Crofer22APU/glass–ceramic/Crofer22APU joints made of both glass–ceramics at room temperature and at 650 °C (lower than *T_g_*), the fracture occurred in the linear mode once the applied stress reached maximum shear strength. This behavior reflects the almost elastic deformation and brittle response of the glass–ceramic joint. However, as expected, at 850 °C (T > *T_g_*), the joints showed enhanced displacement under the applied load and extensive non-linear behavior. This effect is due to the stress relaxation and the softening and viscous flow of the residual glassy phase above *T_g_*, as observed by Zhao et al. [26]. Although both systems were crystallized after the heat treatments during the joining process, the residual glassy phase seemed to be the main factor controlling the mechanical behavior of the joint. As both glass systems were designed for a working temperature of 850 °C, the stress relaxation phenomenon is favorable for reducing the thermal stresses that could be generated due to thermal mismatch at high temperature changes associated with the cell operation. Moreover, the residual glassy phase exhibiting viscous flow could also be beneficial for self-healing and consequently may enhance the long-term stability of the sealant. Chang et al. [12] observed the stress relaxation phenomenon for the as-joined and aged GC-9 glass–ceramic when tested in the temperature range of 650 °C–750 °C, and attributed it to the viscoelastic behavior of the residual glassy phase.

**Figure 5:**
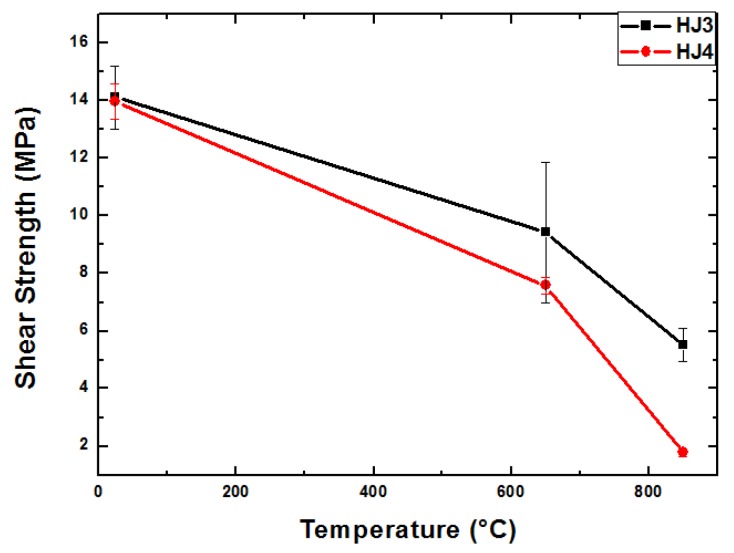
Comparison of shear strength of both glass systems as the function of test temperature.

The shear strength values as determined and calculated from the stress displacement curves shown above (Figure 4) are given in Figure 5. Figure 5 shows the average shear strength obtained from three samples for each composition and at each test temperature. The shear strength of all the joints is seen to decrease with increasing testing temperatures. For the HJ3-based joints the strength reduced from 14.1 (at 25 °C) to 5.5 MPa (850 °C), while for the HJ4-based joints, the shear strength dropped from 13.9 MPa (25 °C) to 1.8 MPa (850 °C). The reduction observed in the strength with increasing temperature was according to expectations and is due to the softening of residual glassy phase at high temperature and its viscous flow (creep deformation). Because of the lower volume fraction of the crystalline phases within the HJ4, its shear strength reduced more drastically compared with the HJ3 glass–ceramic when increasing the testing temperature from 650 °C to 850 °C. Osipova et al. [17] also investigated the shear strength of glass–ceramics at room temperature, 600 °C and 800 °C and found similar behavior of enhanced reduction in shear strength at 800 °C, due to the softening of the remaining glassy phase. 

It is worth highlighting that the HJ3-based joints fractured cohesively (fracture occurred within the glass–ceramic joint), while the HJ4-based joints fractured in an adhesive manner (fracture occurred at the Crofer22APU/glass–ceramic interface) for all the three testing temperatures. 

**Figure 6:**
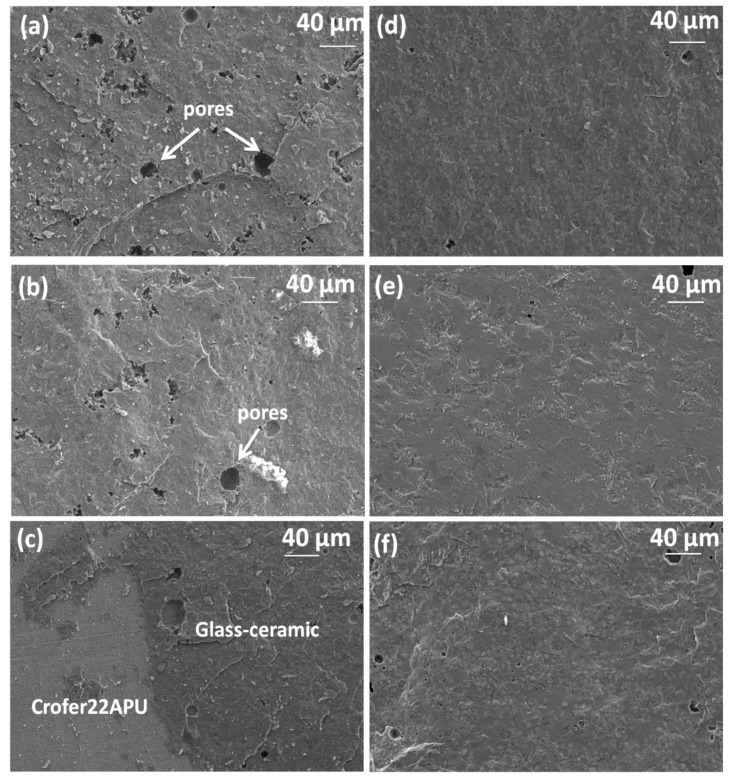
SEM images of top morphology of fracture surfaces of broken joint material; samples (**a**) HJ3 tested at RT, (**b**) HJ3 tested at 650 °C, (**c**) HJ3 tested at 850 °C, (**d**) HJ4 tested at RT, (**e**) HJ4 tested at 650 °C, (**f**) HJ4 tested at 850 °C.

Figure 6 shows the SEM images of the fractured surfaces of the joints based on both the glass systems. For the HJ4-based system, the adhesive fracture resulted in the detachment of the glass–ceramic from one of the joined Crofer22APU plates, therefore, the shown SEM images correspond to the Crofer22APU plate containing all the HJ4 glass–ceramic. The adhesive fracture occurred at one of the two Crofer22APU/HJ4 glass–ceramic interfaces, which resulted in the complete delamination of the HJ4 glass–ceramic at one interface. The corresponding SEM images (Figure 6a,b) of the Crofer22APU/HJ3/Crofer22APU samples tested at room temperature and 650 °C respectively, show that the glass–ceramics completely adhered throughout the joining area of both the Crofer22APU plates. However, for the HJ3 system-based joint tested at 850 °C, the glass–ceramic was also partially detached from one of the Crofer22APU plates, as shown in Figure 6c. The fracture was probably initiated within the glass–ceramic bulk as in the case of low temperature testing, and then propagated to the interface. 

Furthermore, overall thermal expansion coefficient (CTE) of a glass–ceramic, the CTE of individual crystal phases in that glass–ceramic system can play a key role in controlling the fracture behavior as they may create the stress concentration regimes, either within the glass–ceramics and/or at interface.

The HJ3 glass–ceramic has CTE of 10.2 ×10^−6^ K^−1^ that is closely matching CTE of the Crofer22APU (12 ×10^−6^ K^−1^) [27]; however, despite the presence of Sr_2_Al_2_SiO_7_ as main crystalline phase with a CTE of 1.1 ×10^−6^ K^−1^ [28] that could generate localized stresses within the HJ3 glass–ceramic, the mechanical strength was found to be similar to HJ4 system (where Sr_2_Al_2_SiO_7_ is not present).

The HJ4 system has SrSiO_3_ as main crystalline phase having a CTE of 10.9 ×10^−6^ K^−1^ [29], thus reducing localized stresses generation within the glass–ceramic. Anyway, the as-joined HJ4 glass–ceramic has a CTE of 9.3 ×10^−6^ K^−1^ which is lower than that of Crofer22APU, therefore, it can lead to generate stresses at Crofer22APU/HJ4 glass–ceramic interface and make adhesive fracture more favorable under the externally applied load. 

Furthermore, the presence of defects (pores or bubbles etc.) within the glass–ceramics can also lead to initiation of crack. The high degree of devitrification in the HJ3 glass–ceramic increases the possibility to generate residual micro porosity due to enhanced viscosity cause by crystallization. These micro pores could be another possible reason to initiate the crack within the HJ3 glass–ceramic. Hasanabadi et al. [9] also tested the Crofer22APU/glass–ceramic/Crofer22APU joints under torsion shear conditions and found that the presence of pores as major reason for crack initiation. On the other hand, even if the higher amount of the residual glassy phase in the HJ4 glass–ceramic minimizes the porosity due to its viscous behavior, the room temperature mechanical strength is not improved with respect to the HJ3 system, likely due to higher residual thermal stresses at the Crofer22APU/HJ4 glass–ceramic interface. 

The lower mechanical strength for HJ4 with respect to HJ3 at 650 °C could be attributed to the presence of cristobalite (SiO_2_) phase in the HJ4 glass–ceramic [24], that has different polymorphs and cause volume expansion around 250 °C, thus determining micro defects in the glass–ceramic at the interface with Crofer22APU.

The SEM images of fractured surfaces show the presence of micro pores in the HJ3 glass–ceramic (Figure 6a–c); however, the HJ4 glass–ceramic seems denser with slight residual porosity (Figure 6d–f). 

**Figure 7:**
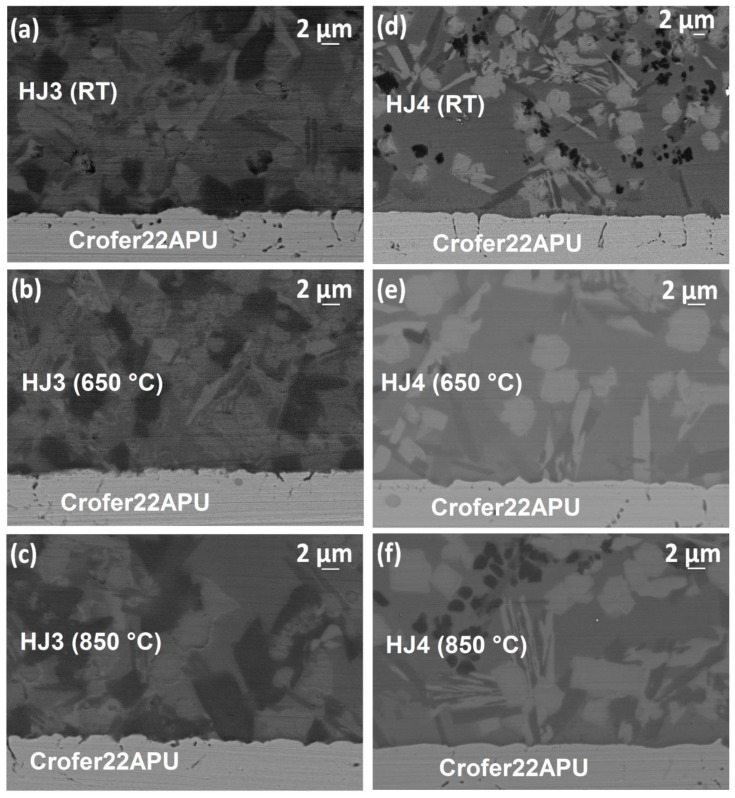
SEM images of interface of Crofer22APU with (**a**) HJ3 tested at RT, (**b**) HJ3 tested at 650 °C, (**c**) HJ3 tested at 850 °C, (**d**) HJ4 tested at RT, (**e**) HJ4 tested at 650 °C, (**f**) HJ4 tested at 850 °C. Details about the microstructure of both the glass–ceramics can be found elsewhere [24].

Figure 7 shows the SEM images of the Crofer22APU/glass–ceramic interface for broken samples for both the glass systems tested at three different temperatures under shear load. For the HJ4 system, the images correspond to the Crofer22APU/HJ4 contained all the joined glass–ceramic well adhered to Crofer22APU surface after the fracture. The SEM images showed no cracks at glass–ceramics/Crofer22APU interfaces tested at room temperature as well as at high testing temperatures, thus demonstrating a good thermomechanical compatibility. A uniform microstructure of both the glass–ceramics was observed. For a particular glass system, the Crofer22APU/glass–ceramic interfaces showed a similar morphology after being tested at three different temperatures. Further details about the microstructure of both the glass–ceramics can be found elsewhere [24].

**Figure 8:**
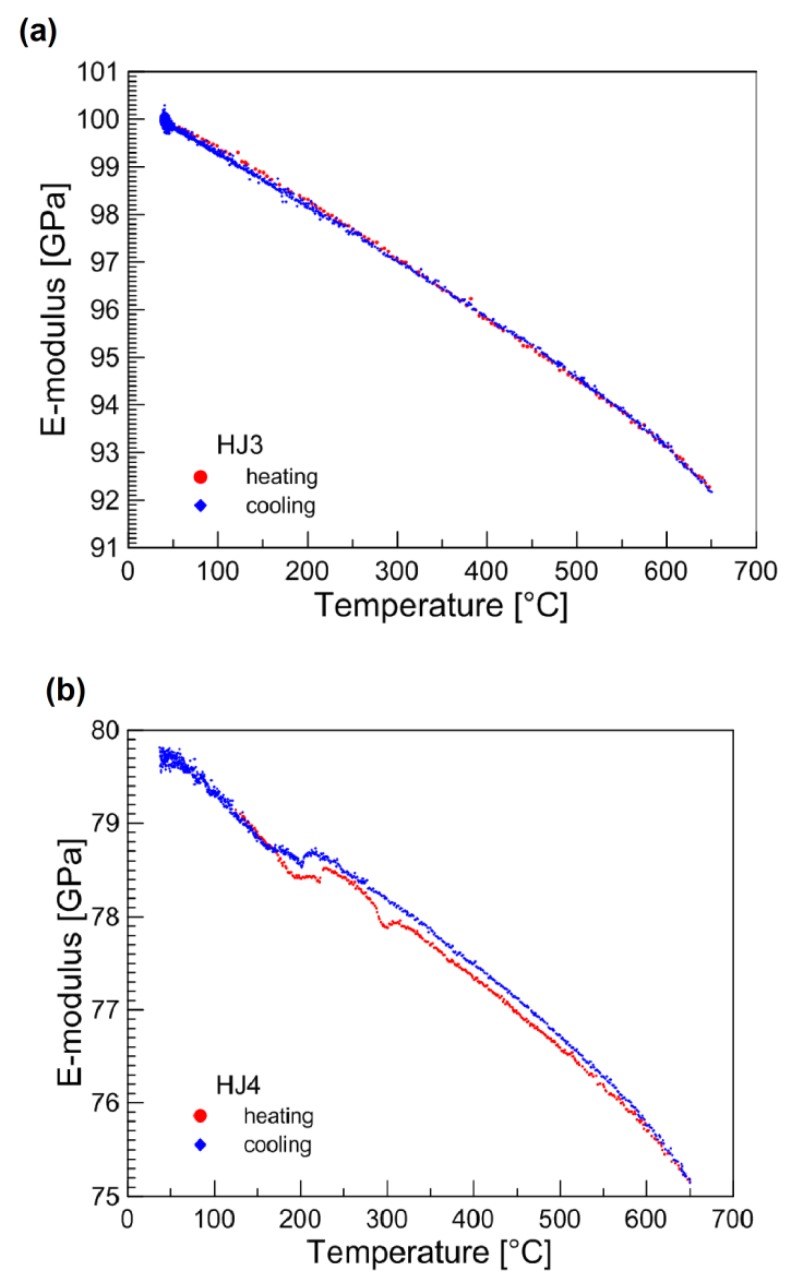
Elastic modulus of as-joined (**a**) HJ3 and (**b**) HJ4 glass–ceramics, measurement was done from room temperature to 650 °C.

The elastic moduli of the as-joined glass–ceramics for both the glass systems are shown in Figure 8. Measurements were performed from room temperature to 650°C and elastic modulus was measured both during the heating and cooling. Above 650 °C, the obtained resonance frequencies were not sufficient to measure the reliable values due to the possible softening of the glass–ceramics. The direct comparison shows that the HJ3 glass–ceramic (Figure 8a) has higher values of elastic modulus as compared with the HJ4 system (Figure 8b). The higher volume fraction of crystals in the HJ3 glass–ceramic made this system stiffer than the HJ4 glass–ceramic. Similar behavior was observed by Milhans et al. [30], where reduced modulus was observed to increase with increasing volume content of the crystalline phases in the glass–ceramics, as measured by nanoindentation. Zhao et al. [31] also compared the elastic modulus of different glass–ceramic systems having different amounts of the residual glassy phases. The glass–ceramic with less quantity of residual glassy phase showed higher elastic modulus. For the HJ3 glass–ceramic, the elastic modulus reduced from 100 GPa (at room temperature) to 92 GPa (650 °C), whereas for the HJ4 glass–ceramic it decreased from 80 GPa (room temperature) to 75 GPa (650 °C). The elastic modulus curve for the HJ4 glass–ceramic (Figure 8) also shows discontinuities around 230°C during heating cycle and around 230 °C–270 °C during cooling. This is due to the presence of cristobalite (SiO_2_) phase in the HJ4 glass–ceramic [24,32]. Nevertheless, the obtained elastic moduli for both the systems (HJ3 and HJ4) are comparable or slightly higher than the elastic modulus of the glass–ceramics available in the literature (50–80 GPa) [8,22,30,31,33,34]. 

## 4. Conclusions

Mechanical testing in shear conditions performed at room temperature, 650 °C and 850 °C enabled to quantify the temperature dependence of the investigated shear parameters. The mechanical strength of both glass–ceramic-joined samples tested at 650 °C was retained up to 60–70% of the RT values, while a substantial decrease was observed at 850 °C (T > *T_g_*).

Similarly, as expected the elastic modulus of both the glass–ceramics also showed nearly linear reduction with temperature. The volume fraction of the crystalline phases has been found to be an important factor controlling the mechanical properties at high temperatures. The higher amount of residual glassy phase in the glass–ceramic reduces the high-temperature mechanical properties of the sealant but promotes the stress relaxation possibilities due to softening, which is beneficial to release the thermal stresses at high temperature.

This study provides insights for design and development of glass–ceramic sealants for SOEC working at 850 °C.
